# Evidence for Mediodorsal Thalamus and Prefrontal Cortex Interactions during Cognition in Macaques

**DOI:** 10.1093/cercor/bhv093

**Published:** 2015-05-15

**Authors:** Philip G. F. Browning, Subhojit Chakraborty, Anna S. Mitchell

**Affiliations:** 1 Glickenhaus Laboratory of Neuropsychology and Friedman Brain Institute, Fishberg Department of Neuroscience, Icahn School of Medicine at Mount Sinai, New York, NY 10029, USA; 2 Department of Bioengineering, Imperial College London, South Kensington, London SW7 2BP, UK; 3 Department of Experimental Psychology, Oxford University, Oxford OX1 3UD, UK

**Keywords:** amnesia, decision-making, dysfunction, learning, monkey, orbitofrontal cortex, prefrontal cortex

## Abstract

It is proposed that mediodorsal thalamus contributes to cognition via interactions with prefrontal cortex. However, there is relatively little evidence detailing the interactions between mediodorsal thalamus and prefrontal cortex linked to cognition in primates. This study investigated these interactions during learning, memory, and decision-making tasks in rhesus monkeys using a disconnection lesion approach. Preoperatively, monkeys learned object-in-place scene discriminations embedded within colorful visual backgrounds. Unilateral neurotoxic lesions to magnocellular mediodorsal thalamus (MDmc) impaired the ability to learn new object-in-place scene discriminations. In contrast, unilateral ablations to ventrolateral and orbital prefrontal cortex (PFv+o) left learning intact. A second unilateral MDmc or PFv+o lesion in the contralateral hemisphere to the first operation, causing functional MDmc–PFv+o disconnection across hemispheres, further impaired learning object-in-place scene discriminations, although object discrimination learning remained intact. Adaptive decision-making after reward satiety devaluation was also reduced. These data highlight the functional importance of interactions between MDmc and PFv+o during learning object-in-place scene discriminations and adaptive decision-making but not object discrimination learning. Moreover, learning deficits observed after unilateral removal of MDmc but not PFv+o provide direct behavioral evidence of the MDmc role influencing more widespread regions of the frontal lobes in cognition.

## Introduction

It is a widely held view that direct cortico-cortical connections convey perceptual, motor, and cognitive information, whereas it is customary to think of the thalamus as a structure relaying sensorimotor information (Sherman and Guillery 2002). However, mounting evidence shows that some thalamic nuclei (e.g., mediodorsal thalamus via cortico-thalamo-cortical links) may modulate information transfer across cortical areas and, via these interconnections may influence cognition and behavior in their own right (Jones 2007; Sherman and Guillery 2011; Wurtz et al. 2011; Mitchell et al. 2014).

The mediodorsal thalamus in primates and rodents has several distinct subdivisions, each with its own unique anatomical links with other brain regions (Mitchell and Chakraborty 2013). Specifically, the anatomy pertaining to the magnocellular subdivision of the mediodorsal thalamus (MDmc) involves reciprocal and crossed interconnections with lateral and medial orbital prefrontal cortex (Preuss and Goldman-Rakic 1987; Ray and Price 1993). There are also non-reciprocal inputs to MDmc from medial (areas 25 and ventral 32) and ventrolateral (45/47/12) subdivisions of prefrontal cortex (Goldman-Rakic and Porrino 1985; Giguere and Goldman-Rakic 1988; Ray and Price 1993; Haber and McFarland 2001) and from other forebrain regions that include entorhinal and perirhinal cortex, amygdala, substantia innominata, and ventral pallidum (Aggleton and Mishkin 1984; Russchen et al. 1987; Ray and Price 1993). In vivo human brain imaging has confirmed via white fiber tract tracing that similar mediodorsal thalamus and prefrontal cortex interconnections exist in humans (Klein et al. 2010). Patients with thalamic damage that includes mediodorsal thalamus report problems with memory and learning new information, and they have impaired cognitive control (Daum and Ackermann 1994; Kopelman et al. 1997; Van der Werf et al. 2003; Zoppelt et al. 2003; Pergola and Suchan 2013). Consequently, the neuronal signals flowing to and from MDmc suggest that it plays a critical role in cognition.

Causal behavioral evidence from monkey models of amnesia demonstrates that after bilateral damage to MDmc, monkeys are impaired in learning new object-in-place scene discriminations, although retention of similar discriminations learnt prior to brain injury is left intact (Mitchell, Baxter et al. 2007; Mitchell and Gaffan 2008). This evidence suggests a distinct function for MDmc in supporting acquisition of new visual information rather than in memory retention. The object-in-place scene discrimination task is an associative learning task combining “what,” “where,” and contextual information with reward for choosing the correct object (Gaffan 1994; Aggleton et al. 2000; Murray and Wise 2010). In monkeys, selective bilateral damage to orbitofrontal cortex, ventrolateral prefrontal cortex, fornix, anterior thalamic nuclei, mammillary bodies, or contralateral disconnection of inferotemporal cortex from prefrontal cortex causes deficits in learning new object-in-place scene discriminations (Parker and Gaffan 1997a; 1997b; Browning et al. 2005; Wilson et al. 2007; Baxter et al. 2007, 2008). Furthermore, bilateral damage to MDmc impairs performance on the object-in-place scene discrimination task (Gaffan and Parker 2000; Mitchell, Baxter et al. 2007). Given this evidence, we had proposed that when MDmc is ablated, the lost relay functions cause disruption to normal communication across the cortex during learning using this object-in-place scene discrimination task (Mitchell, Baxter et al. 2007; Mitchell et al. 2014). Yet, it remains to be determined whether functional interactions of MDmc and interconnected prefrontal cortex are critical during learning in the object-in-place scene discrimination task.

Furthermore, consistent evidence also supports a role for the mediodorsal thalamus in adaptive decision-making in primates (Mitchell, Browning et al. 2007) and in rodents (Corbit et al. 2003; Mitchell and Dalrymple-Alford 2005; Ostlund and Balleine 2008; Bradfield et al. 2013; Parnaudeau et al. 2015). More recently in primates, Izquierdo and Murray (2010) demonstrated that the integrity of a neural network linking the MDmc with the orbital frontal cortex and amygdala is critical for optimal decision-making using the satiety devaluation paradigm. However, the specific functional contributions of the MDmc–PFC interactions have not yet been established in this adaptive decision-making task.

Thus, the present set of experiments assessed the combined contribution of the interactions between MDmc and interconnected ventrolateral and orbitofrontal cortex (PFv+o) during cognition using a disconnection lesion design. Preoperatively, monkeys learned to discriminate between 2 target objects (only 1 was rewarded) embedded within colorful visual background scenes (object-in-place scene discriminations). After unilateral lesions to MDmc or PFv+o, the ability to learn new object-in-place scene discriminations was evaluated. Then, after a second unilateral MDmc or PFv+o neurosurgery to disconnect the contralateral hemisphere (causing functional disconnection of gray matter regions in MDmc and PFv+o in contralateral hemispheres), further learning of new object-in-place scene discriminations was evaluated. If MDmc–PFv+o interactions are critical for new learning, then the contralateral disconnection will induce deficits during learning in the task. The effects of these contralateral lesions were compared with monkeys with ipsilateral disconnection of the MDmc and PFv+o (a control lesion that maintains 1 intact hemisphere). Further cognitive testing of rewarded object discrimination learning and adaptive decision-making using the object-food satiety devaluation paradigm assessed the generality and robust nature of any postoperative deficits.

## Materials and Methods

### Subjects

In the current study, there were 6 rhesus monkeys (*Macaca mulatta*; 1 female [MP2]) aged between 3.2 and 5 years at the beginning of behavioral training. The female monkey (MP2) had been an unoperated control animal in a previous study involving cognitive testing in the computerized apparatus used in the current experiments. Three monkeys (MP1, MP2, and MP3) received contralateral hemisphere disconnection of MDmc from PFv+o (CONTRA); 2 monkeys (MP4 and MP5) received ipsilateral hemisphere disconnection of MDmc from PFv+o (IPSI); and 1 monkey (MP6) received contralateral hemisphere disconnection of midline thalamic nuclei from PFv+o (MidxPFv+o). All experimental procedures were performed in compliance with the United Kingdom Animals (Scientific Procedures) Act of 1986. A Home Office (UK) Project License obtained after review by the University of Oxford Animal Care and Ethical Review committee licensed all procedures. The animals were socially housed together in same sex groups of between 2 and 6 animals. The housing and husbandry were in compliance with the guidelines of the European Directive (2010/63/EU) for the care and use of laboratory animals.

### Apparatus

The computer-controlled test apparatus was identical to that previously described (Mitchell, Browning et al. 2007). Briefly, monkeys sat in a transport box fixed to the front of a large touch-sensitive color monitor that displayed the visual stimuli for all of the experiments. Monkeys reached out through the bars of the transport box to respond on the touchscreen and collect their food reward pellets from a hopper that were automatically dispensed by the computer. Monkeys were monitored remotely via closed circuit cameras and display monitors throughout the testing period.

### Procedures

**Figure 1.**
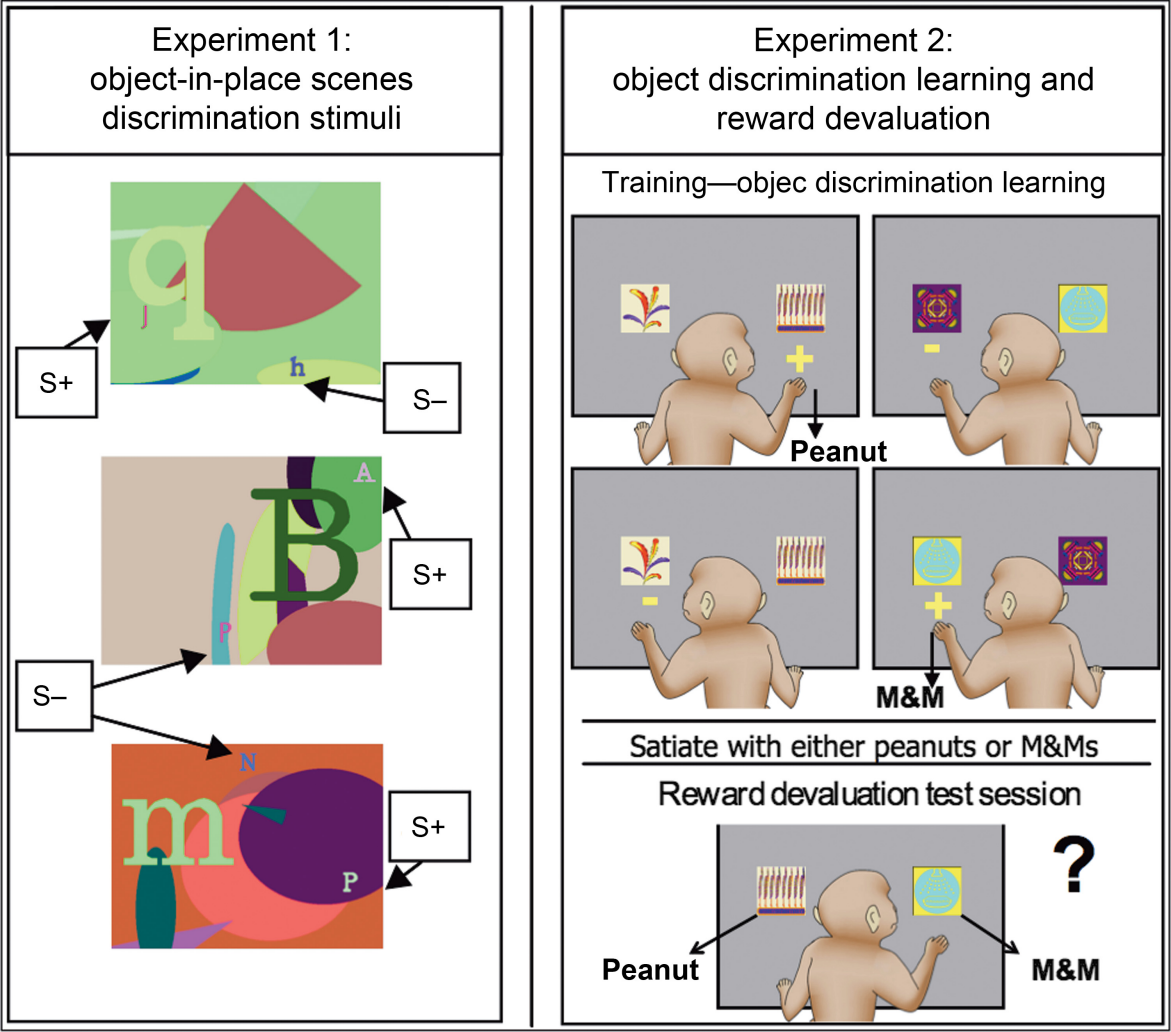
Left: Three examples of object-in-place discrimination stimuli “scenes” used in Experiment 1 in this study. Monkeys respond to each “scene” by touching 1 of the 2 foreground objects. One of the 2 foreground typographic objects in each “scene” denoted by “S+” is arbitrarily designated as correct (reward). The “S−” indicates the locations of the unrewarded foreground objects in each “scene.” The locations and identities of the foreground objects are fixed within each scene but vary across scenes. Right: Object discrimination learning and food devaluation paradigm. During training (object discrimination learning), monkeys are presented with 2 clipart images per trial (60 pairs) and learn which object rewards them with a peanut or an M&M or no reward. After reaching criterion, food reward devaluation is conducted. Monkeys are satiated with 1 food reward just prior to the test session. During devaluation test sessions, pairs of rewarded objects only are presented (30 pairs in total) and the monkey chooses between the 2 objects to receive either a peanut or an M&M reward.

Figure 1 provides a visual model of the 2 experiments incorporating the 3 cognitive tasks and order of testing for the monkeys.

#### Experiment 1: Object-in-Place Scene Discrimination Learning Task

This object-in-place scene discrimination learning task was adapted (Gaffan 1994). Briefly, each trial consisted of an artificially constructed “scene.” There were 2 foreground “objects” for each discrimination, one correct (rewarded) and the other incorrect (non-rewarded), consisting of randomly selected small-colored typographic characters each placed in a constant location. Each discrimination scene was unique in that they varied in several randomly selected attributes including (1) the background color of the screen, (2) the location of ellipses on the screen, (3) the color, size, and orientation of ellipse segments, (4) the typographic character, clearly distinct in size from the foreground objects, and (5) the color of the typographic character. All the colors were assigned with the constraint that the foreground objects should be visible (that is, there was a minimum separation in color space between the colors of a foreground object and the color of any element of its local background).

##### Behavioral Training

Pretraining followed a previously published protocol (Mitchell, Baxter et al. 2007). The behavioral training began once the monkeys were reliably touching the foreground objects when presented with a new scene and completing 50 trials a day with minimal accuracy errors (i.e., touching any location on the screen other than the foreground typographic characters). Problems were introduced with 2 foreground objects (one correct and one incorrect, as described earlier), and the number of scenes given in each session was gradually increased, based on each monkey's performance. The locations and identities of the foreground objects were fixed within each scene but varied between scenes. As these scenes were randomly generated, an infinite number of unique scenes could be presented. A touch to the correct object caused the object to flash for 2 s whereas the incorrect object stayed on screen along with the colorful visual background scene, then the screen blanked and a reward pellet was delivered into the hopper. A touch to the incorrect object caused the screen to blank immediately, no reward was given, and an ITI of 10 s followed before the start of the next trial. For the first presentation of the list of novel scenes only, incorrect responses were followed by a correction trial in which the scene was re-presented with only the correct object present. Touches anywhere else in the scene caused the screen to blank, and the trial was repeated.

In the final version of the task, the monkey was required to learn a novel set of 20 new object-in-place scene discriminations within each testing session (or 10 novel scenes for MP4 as this monkey could not maintain stable performance while learning 20 new scenes each day), by being exposed to an initial run through the set of 20 (or 10 for MP4) discriminations followed by 7 repetition trials of the set of 20 (or 10 for MP4) discriminations within the session (in the same order in each of the repetitions through the set of discriminations). On the next daily testing session, a novel set of 20 (or 10 for MP4) discriminations was presented and learnt within the session in the same fashion as mentioned above, and so on. During daily learning, performance on the first presentation of the discriminations (Trial 1) is at chance, as the monkey has no information about which is the correct object to choose on the very first exposure of each discrimination. Then, through subsequent repetitions of the same discriminations within the session (Trials 2–8), a monkey learns the discriminations rapidly. Once stable learning is established within each session across several weeks of testing with new discrimination problems presented in each testing session, a monkey has a rest period of 2 weeks (equivalent in duration to a “postoperative rest”) then a preoperative performance test for 13 days is conducted. For Days 1 and 2 of the performance test, the monkey receives 1 session of 10 (or 5 for MP4) novel object-in-place scene discriminations (with 8 repetition trials within the session), again with novel discriminations used each day. Then, for Days 3–13, the monkey receives their preoperative performance test with 20 (or 10 for MP4) novel discriminations each day and 8 repetition trials within each session. The preoperative performance test data were analyzed from the sessions completed on Days 4–13. After surgery and 2 weeks postoperative rest, an identical method for the postoperative performance test was followed to obtain postoperative within-session learning data from the last 10 postoperative sessions (Days 4–13). Proficiency in preoperative (Pre) and postoperative (Post1) within-session learning in this task is expressed as average percentage errors in repetition trials 2–8 across the final 10 sessions of testing (i.e., Days 4–13). After the first postoperative performance test was complete, all monkeys in the current study received their second disconnection neurosurgery. After at least 2 weeks of postoperative recovery from the second neurosurgery, all monkeys in the current study completed another postoperative performance test for 13 days as detailed earlier.

#### Experiment 2: Object Discrimination Learning and Reinforcer Devaluation

The automated task and stimuli were identical to the previously published procedure (Mitchell, Browning et al. 2007). After the disconnection neurosurgery and completion of the second postoperative performance test in Experiment 1, all monkeys with contralateral or ipsilateral disconnections started learning the same set of distinct pairs of clipart objects as 2-choice object discrimination learning problems with each pair representing 1 object discrimination problem (60 problems in total). Each trial began with the presentation of 1 pair of clipart images against a gray background, one on the left side of the screen and one on the right, and these positions were pseudo-randomized across trials. One object was arbitrarily assigned correct in each pair. Touching the correct object caused it to flash for 2 s (the incorrect object immediately disappeared), and it also resulted in the immediate delivery of a reward of either a half-peanut or an M&M chocolate candy into the food hopper. Half of the rewarded objects delivered a half-peanut, and the other rewarded objects resulted in an M&M. Each problem appeared once in each session. Touches to the incorrect object caused both objects to disappear immediately, and no reward was delivered. The ITI was 30 s after a choice was made, and a touch to the screen during the ITI reset it. Training continued until reaching a criterion of 270 or more correct responses over 5 consecutive sessions (90% or greater correct).

##### Reward Devaluation

On reaching criterion, a series of sessions of critical trials were presented in which the 60 rewarded objects were randomly assigned to create 30 pairs of critical trials, each pair of trials offering a choice between a peanut-rewarded object and an M&M-rewarded object (i.e., there were no non-rewarded objects presented). Some sessions of critical trials were preceded by a devaluation procedure in which the monkey was allowed to consume 1 of the 2 food rewards to satiation before beginning the critical trial session. The sequence of critical trial sessions was baseline, peanut devaluation, baseline, and M&M devaluation (Test 1). The same sequence was repeated once (Test 2). Each critical trial session was separated by at least 1 standard training session (as mentioned above), and monkeys had at least 2 days of rest after a reward devaluation critical trial session. For the reward devaluation critical trial sessions, the monkey sat in their transport box in a separate, familiar room, and a plastic box was fixed to the front of the cage containing a known amount of food reinforcer (either M&Ms or peanuts). The monkey was left undisturbed for 15 min to consume this food. If the food was completely eaten, the box was refilled. The monkey was then observed closely, and once it had not taken any food for 5 min, the box was removed from the cage. Then, when the monkey's cheek pouches were not visibly full with food, it was moved to the testing cubicle and the critical food devaluation trial session begun. It is important to note that, during these critical devaluation trial sessions when the peanuts or M&Ms have been satiated, no further learning of object–reward associations can occur because each pair of objects followed by a food reward is only presented once during the critical devaluation session. The critical measure of performance was a score composed of the difference in a number of choices of objects paired with a particular food on critical baseline sessions and on critical devaluation sessions preceded by the selective satiation of that food being devalued. These scores were added together for each devalued food. This was calculated separately for each sequence of critical trial sessions (Test 1 and Test 2), and the mean was taken as the overall score. Higher positive difference scores indicate sensitivity to reinforcer devaluation.

### Surgery

All 6 monkeys in the current study had 2 neurosurgeries each (see Table 1 for details of the order of lesions for each animal). In the first unilateral neurosurgery, monkeys that received neurotoxic injections to MDmc were as follow: MP1 (left sided), MP2 (right sided), and MP4 (right sided), and monkeys that received ablations of PFv+o were as follows: MP3 (right sided) and MP5 (left sided). Monkey MP6 received neurotoxic injections in the ventral midline thalamic nuclei (right sided). In the second unilateral neurosurgery, monkeys that received unilateral neurotoxic injections into MDmc were as follows: MP3 (left sided) and MP5 (left sided) and monkeys that received unilateral ablation to PFv+o were as follows: MP1 (right sided), MP2 (left sided), MP4 (right sided), MP5 (left sided), and MP6 (left sided). Therefore, monkeys with the contralateral hemisphere disconnection of MDmc from PFv+o (CONTRA, *n* = 3) were as follows: MP1, MP2, and MP3. Monkeys with the ipsilateral hemisphere disconnection of MDmc from PFv+o (IPSI, *n* = 2) were as follows: MP4 and MP5. Monkey MP6 received a contralateral hemisphere disconnection of ventral midline thalamus from PFv+o (*n* = 1).

**Table 1:** Preoperative and postoperative performance test scores of individual monkeys during object-in-place discrimination learning (Experiment 1) with additional single case analyses (Crawford and Howell 1998; Crawford and Garthwaite 2002) comparing performance of each individual animal at each lesion stage with the normative preoperative performance data (Mean = 6.75, SD = 3.1, *n* = 6)

Monkey	Pre	Post1—thalamic	Thalamic single case	Post1—UniPFv+o	PFv+o single case	Post2—CONTRA	CONTRA single case	Post2—IPSI	IPSI single case
MP1 (CONTRA)	6.36	23.14	0.004			35.43	0.001		
MP2 (CONTRA)	3.43	19.00	0.015			30.07	0.001		
MP3 (CONTRA)	6.21			13.36	0.11	26.07	0.002		
MP4 (IPSI)	12.43	29.57	0.001					14.14	0.08
MP5 (IPSI)	4.86			5.43	0.71			7.57	0.82
MP6 (MidxPFv+o)	7.21	(8.29)	0.66			(5.43)	0.71		
Mean Preop	6.75								
Mean UniMDmc		23.90							
Mean UniPFv+o				8.07					
Mean CONTRA						30.52			
Mean IPSI								10.86	

Note: Actual *P*-values reported in italics at two-tailed probability.

Neurosurgical procedures were performed in a dedicated operating theater under aseptic conditions and aided by an operating microscope. Steroids (methylprednisolone, 20 mg/kg) were given the night before surgery intramuscularly (i.m.), and 4 doses were given 4–6 h apart (intravenously [i.v.] or i.m.) on the day of surgery to protect against intraoperative edema and postoperative inflammation. Each monkey was sedated on the morning of surgery with both ketamine (10 mg/kg) and xylazine (0.25–0.5 mg/kg, i.m.). Once sedated, the monkey was given atropine (0.05 mg/kg, i.m.) to reduce secretion, antibiotic (amoxicillin, 8.75 mg/kg) as prophylaxis against infection, opioid (buprenorphine 0.01 mg/kg, repeated twice at 4- to 6-h intervals on the day of surgery, i.v. or i.m.) and nonsteroidal anti-inflammatory (meloxicam, 0.2 mg/kg, i.v.) agents for analgesia, and an H2 receptor antagonist (ranitidine, 1 mg/kg, i.v.) to protect against gastric ulceration as a side effect of the combination of steroid and nonsteroidal anti-inflammatory treatment. The head was shaved and an intravenous cannula put in place for intraoperative delivery of fluids (warmed sterile saline drip, 5 mL/h/kg). The monkey was moved into the operating theater, intubated, placed on sevoflurane anesthesia (1–4%, to effect, in 100% oxygen), and then mechanically ventilated. A hot air blower (Bair Hugger) allowed maintenance of normal body temperature during surgery. Heart rate, oxygen saturation of hemoglobin, mean arterial blood pressure, tidal CO2, body temperature, and respiration rate were monitored continuously throughout the surgery.

#### MDmc Lesions

The procedure for performing the MDmc neurotoxic lesions has been described in detail elsewhere (Mitchell, Browning et al. 2007). Briefly, the monkey was placed in a stereotaxic head holder and the head cleaned with alternating antimicrobial scrub and alcohol and draped to allow a midline incision. After opening the skin and underlying galea in layers, a large D-shaped bone flap was created in the cranium over the area of the operation and the dura over the posterior part of the hemisphere was cut and retracted to the midline. Veins draining into the sagittal sinus were cauterized and cut. The hemisphere was retracted with a brain spoon, and the splenium of the corpus callosum was cut in the midline with a glass aspirator. The tela choroidea was cauterized at the midline, posterior, and dorsal to the thalamus using a metal aspirator that was insulated to the tip. The posterior commissure, the third ventricle posterior to the thalamus, and the most posterior 5 mm of the midline thalamus were exposed. Stereotaxic coordinates were set from the posterior commissure at the midline using the third ventricle as a guide by positioning a stereotaxic manipulator holding a blunt tipped 26-gauge needle of a 10 μL Hamilton syringe above this site. The monkey brain atlas (Ilinsky and Kultas-Ilinsky 1987) was used to calculate the coordinates of the intended lesion site. Neurotoxic unilateral injections to the intended dorsal thalamic nuclei in subjects MP1-6 were produced by 5 × 1 μL injections of a mixture of ibotenic acid (10 mg/mL; Biosearch Technologies) and NMDA (10 mg/mL) dissolved in sterile 0.1 mm PBS. This mixture of ibotenic acid and NMDA targets NMDA receptors and metabotropic glutamate receptors and has previously produced excellent thalamic lesions in rhesus macaques (Mitchell, Baxter et al. 2007; Mitchell, Browning et al. 2007; Mitchell et al. 2008; Mitchell and Gaffan 2008). The needle was positioned for the first set of coordinates: anteroposterior (AP), +5.2 mm anterior to the posterior commissure; mediolateral (ML), +1.2 mm lateral to the third ventricle; dorsoventral (DV), −4.0 mm (to compensate for the hole positioned 1 mm above the tip of the needle) ventral to the surface of the thalamus directly above the intended lesion site. Each injection was made slowly over 4 min, and the needle was left in place for 4 min before being moved to the next site. The needle was then repositioned for the second set of coordinates: AP, +4.2 mm; ML, +1.5 mm; and DV, −5.0 mm. The third, fourth, and fifth sets of coordinates were AP, +4.2 mm, ML, +1.5 mm, and DV, −3.0 mm; AP, +3.4 mm, ML, +1.7 mm, and DV, −4.0 mm; and AP, +3.4 mm, ML, +1.7 mm, and DV, −3.0 mm, respectively. In each case, the DV coordinate was relative to the surface of the thalamus at the injection site. For MP6, the same AP and ML coordinates (as mentioned above) were used, whereas the DV coordinates extended ventrally a further −10.0 mm in the first set of coordinates prior to making the neurotoxic injections by mistake. When the lesion was complete, the dura was repositioned but not sewn, the bone flap was replaced and held with loose sutures, and the galea and skin were closed with sutures in layers. To reduce cerebral edema, mannitol (20%; a sugar alcohol solution; 1 mg/kg, i.v.) was administered slowly for 30 min whereas the monkey was still anesthetized. Then, the monkey was removed from the head-holder and anesthesia discontinued. The monkey was extubated when a swallowing reflex was observed, placed in the recovery position in a cage within a quiet, darkened room, and monitored continuously. Normal posture was regained upon waking (waking times varied between 10 and 40 min after the discontinuation of the anesthesia); all monkeys were kept warm with blankets during this time. The morning after surgery, the monkey was moved to a separate cage within their homeroom enclosure. Operated monkeys re-joined their socially housed environment as soon as practical after surgery, usually within 3 days of the operation.

#### PFv+o Lesions

The monkey was placed in a standard head-holder and the head cleaned with alternating antimicrobial scrub and alcohol and draped to allow a midline incision. Then, the skin and the underlying galea were opened in layers, and a bone flap was created over the frontal lobes; the craniotomy was extended with rongeurs as necessary. The dura was cut and reflected over the frontal lobe, and veins draining into the sagittal sinus were cauterized and cut. The lesion extended from the ventral lip of the principal sulcus ventrally to include the fundus of the lateral orbital sulcus and around to the fundus of the rostral sulcus, posteriorly it extended to a line joining the posterior tip of the principal sulcus and the anterior tip of the inferior limb of the arcuate sulcus, then extending from the tip of the arcuate sulcus to the posterior tip of the lateral orbital sulcus. The anterior and posterior limits within the orbital frontal cortex were lines joining the tips of the lateral and medial orbital sulci, extending medially to the midline. All the cortex was removed within these limits by subpial aspiration using a small-gauge sucker insulated everywhere except at the tip; electrocautery was applied to remove the pia mater and control bleeding encountered during the ablation. When the lesion was complete, the dura was sewn over the lesion site, the bone flap was replaced and held with loose sutures, and the skin and galea were closed in layers. The monkey was then removed from the head-holder and anesthesia discontinued. The monkey was extubated when a swallowing reflex was observed, returned to a separate recovery cage within their homeroom enclosure, and monitored continuously. Normal posture was regained usually within 10–20 min; all monkeys were kept warm with blankets during this time. Operated monkeys re-joined their socially housed environment as soon as practical after surgery, usually within 2–3 days of the operation.

After all neurosurgery, each monkey was monitored continuously for at least 48 h. Postoperative medication continued in consultation with veterinary staff, including steroids (dexamethasone, 1 mg/kg, i.m.); for MDmc lesions, the dose was once every 12 h for 4 days, then once every 24 h for 3 days; for PFv+o lesions, the dose was once every 12 h for 3 days, then once every 24 h for 2 days; analgesia (buprenorphine, 0.01 mg/kg, i.m.) for 48 h; and antibiotic treatment (amoxicillin, 8.75 mg/kg, oral) for 5 days. Gastric ulcer protection (omeprazole, 5 mg/kg, oral; and antepsin, 500 mg/kg, oral) commenced 2 days prior to surgery and continued postoperatively for the duration of other prescribed medications, up to 7 days.

### Histology

After completion of all behavioral testing, each monkey was sedated with ketamine (10 mg/kg), deeply anesthetized with intravenous barbiturate and transcardially perfused with 0.9% saline followed by 10% formalin. The brains were cryoprotected in formalin-sucrose and then sectioned coronally on a freezing microtome at 50 μm of thickness. A 1-in-10 series of sections was collected throughout the cerebrum that was expanded to a 1-in-5 series throughout the thalamus. All sections were mounted on gelatin-coated glass microscope slides and stained with cresyl violet.

### Statistical Analysis

The data from Experiment 1 were separated into different analyses linked to the stages of each unilateral neurosurgery. Repeated-measures *t*-tests with significance set at *P* < 0.05 were used for preoperative versus postoperative performance comparisons for each of the different surgical lesion groups. The data analysis from Experiment 2 included data from the CONTRA (*n* = 3) and IPSI (*n* = 2) groups of monkeys. Nonparametric statistics were used to compare the mean number of errors and mean number of sessions to criterion during learning the object discrimination task, and the mean difference scores (Test 1, Test 2, and Overall difference score) during the food reward devaluation critical test sequences.

## Results

### Assessment of MDmc Lesions

**Figure 2.**
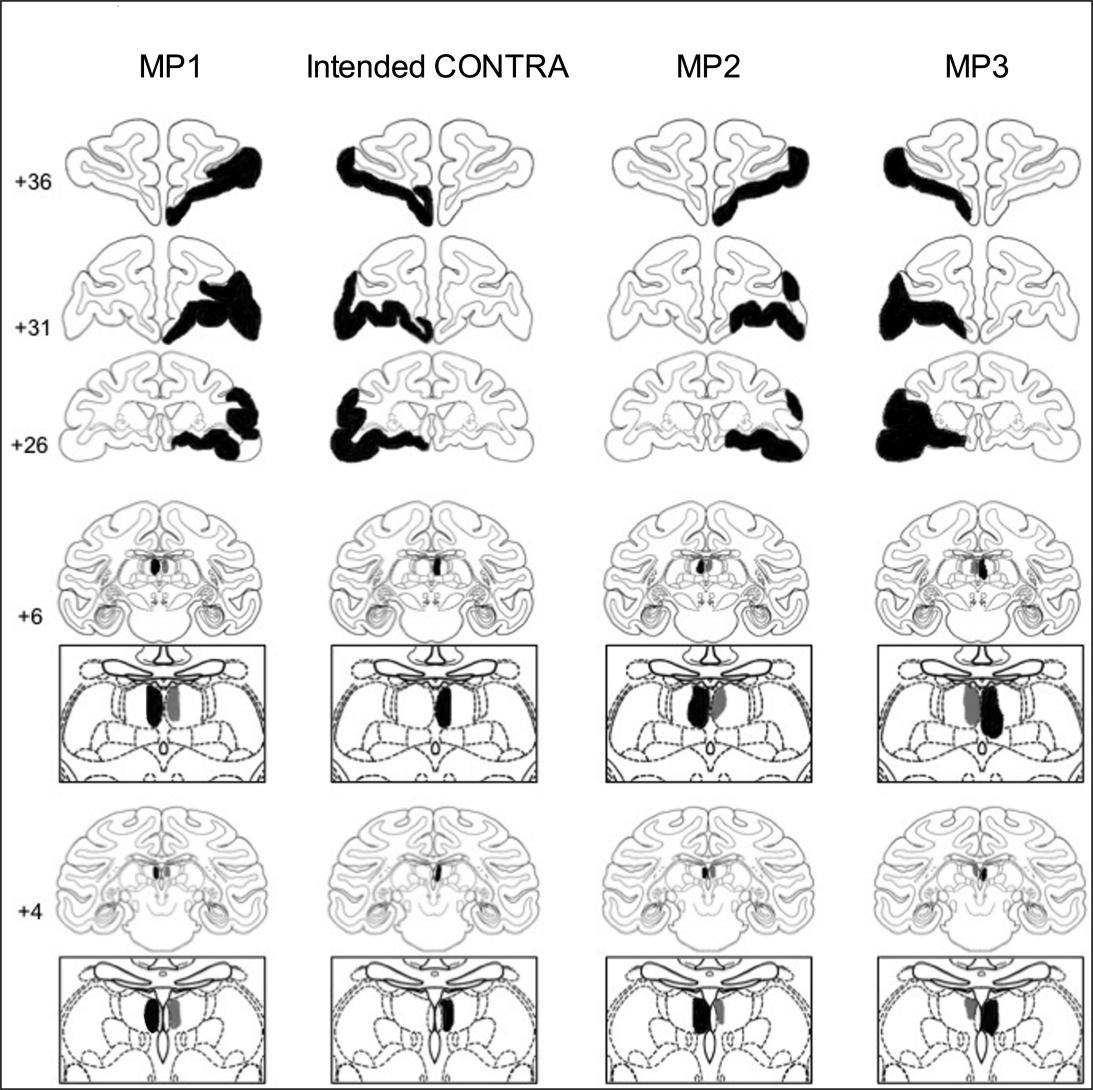
Schematic coronal sections from a standard rhesus monkey (Saunders 2006) showing an example of the intended contralateral hemisphere disconnection of MDmc–PFv+o (CONTRA, second column) and in Columns 1, 3, and 4 in black ink, the reconstructed estimated damage after unilateral PFv+o ablations and unilateral neurotoxic injections into the MDmc of 3 monkeys (MP1, MP2, and MP3) with contralateral hemisphere disconnection. The ipsilateral retrograde degeneration within MD caused by the PFv+o ablation is depicted in gray. Numbers refer to the coronal sections from the atlas.

**Figure 3.**
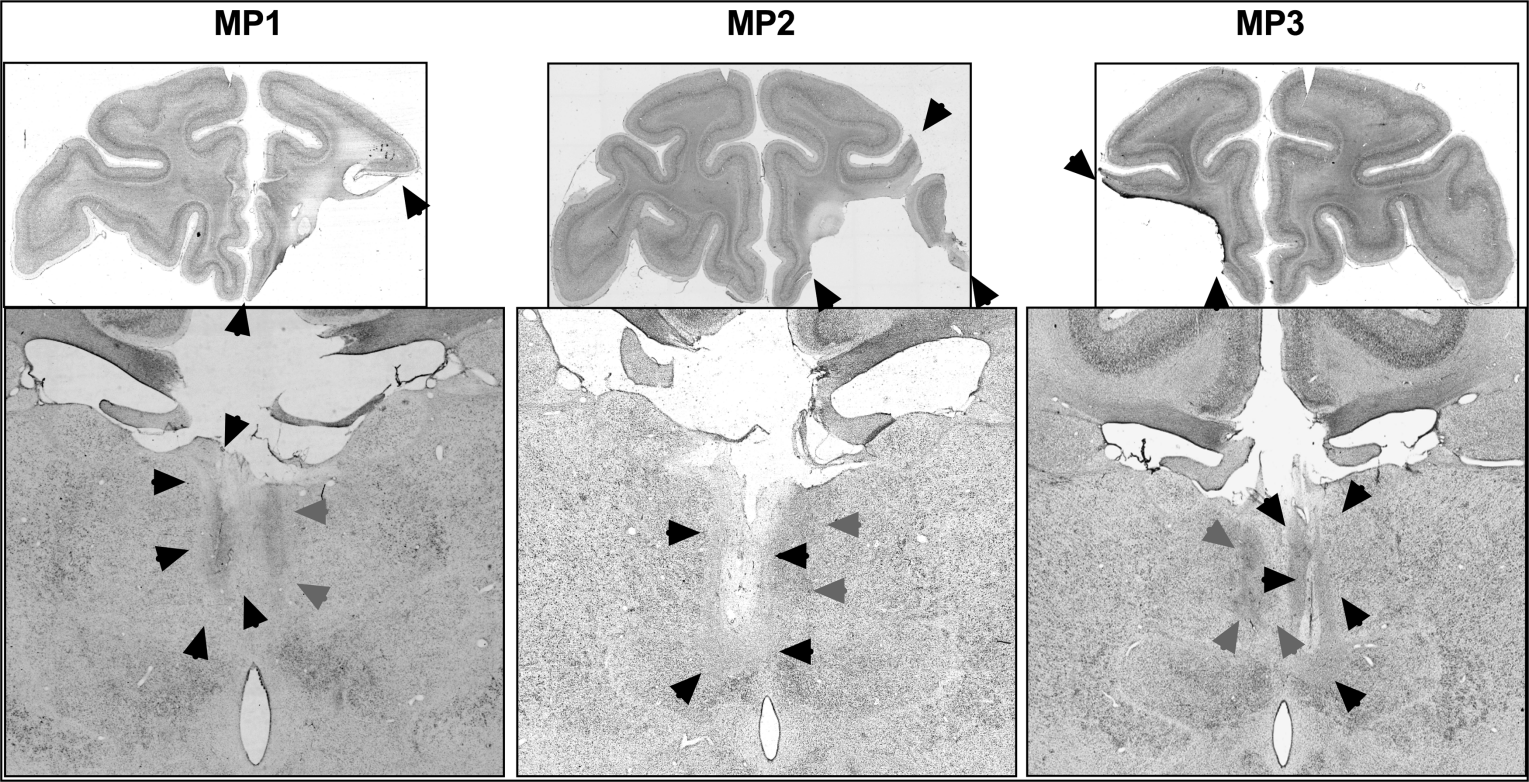
Top: Photomicrographs of a coronal slice at the level of IA+31 showing the unilateral damage (black arrows) to the PFv+o in monkeys, MP1 (first column), MP2 (second column), and MP3 (third column). Bottom: Photomicrographs of a coronal slice at the level of IA+6 for monkeys, MP1, MP2, and MP3 with contralateral hemisphere disconnections, showing the unilateral damage (black arrows) from the MDmc neurotoxic injections and retrograde degeneration (gray arrows) caused by unilateral PFv+o ablations in the ipsilateral hemisphere of MD.

**Figure 4.**
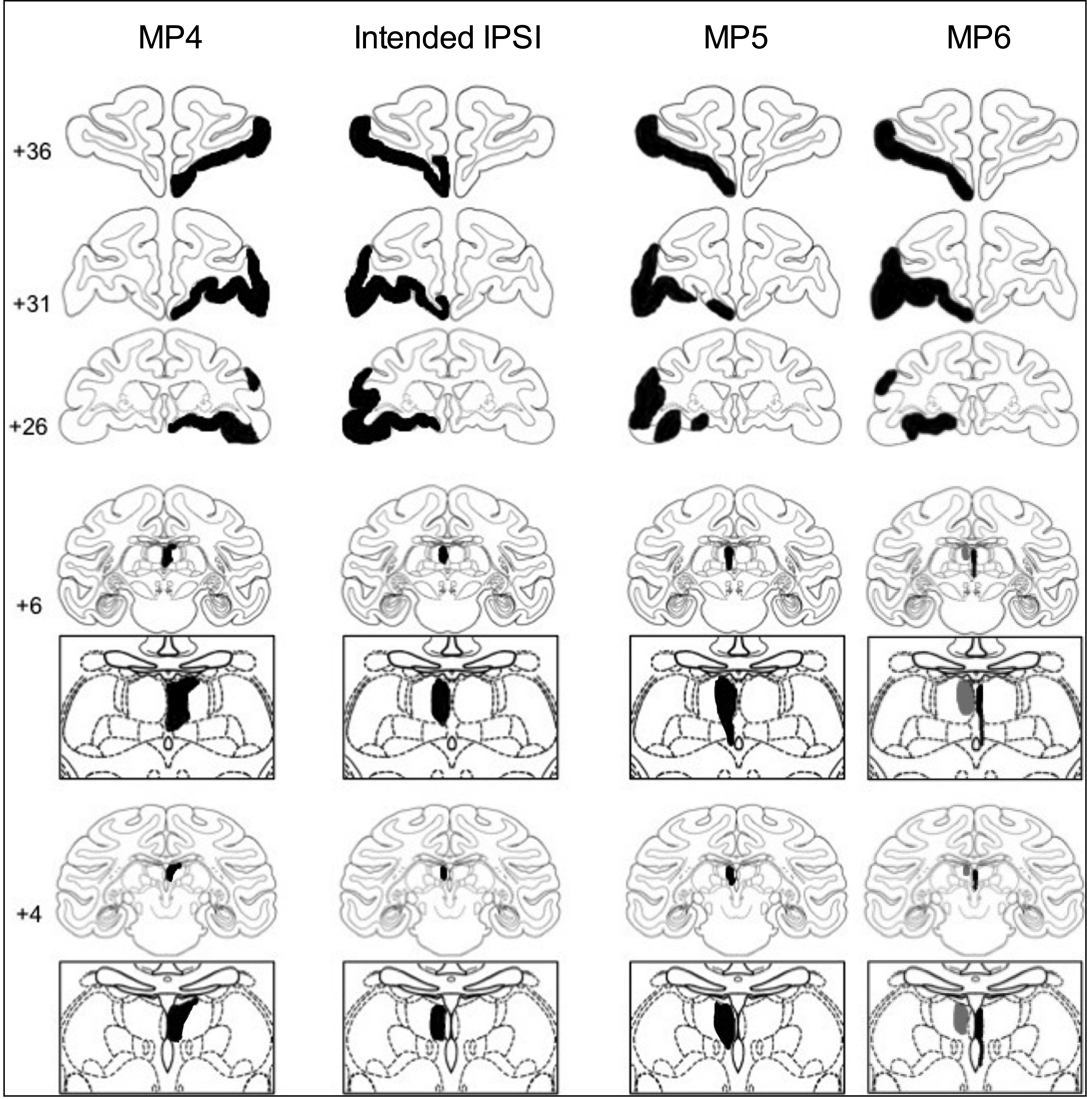
Schematic coronal sections from a standard rhesus monkey (Saunders 2006) showing an example of the intended ipsilateral hemisphere disconnection of the MDmc–PFv+o (IPSI, second column) and in Columns 1 and 3 in black ink, reconstructed estimated damage after unilateral PFv+o ablations and unilateral neurotoxic injections into the MDmc of the 2 monkeys (MP4 and MP5) with ipsilateral hemisphere disconnection in black. In column 4 in black ink is reconstructed estimated damage for monkey MP6 after left hemisphere unilateral PFv+o ablations and right hemisphere unilateral neurotoxic injections into the midline and ventral thalamic nuclei. The ipsilateral retrograde degeneration caused by left hemisphere PFv+o ablation is depicted in gray within the left MD.

**Figure 5.**
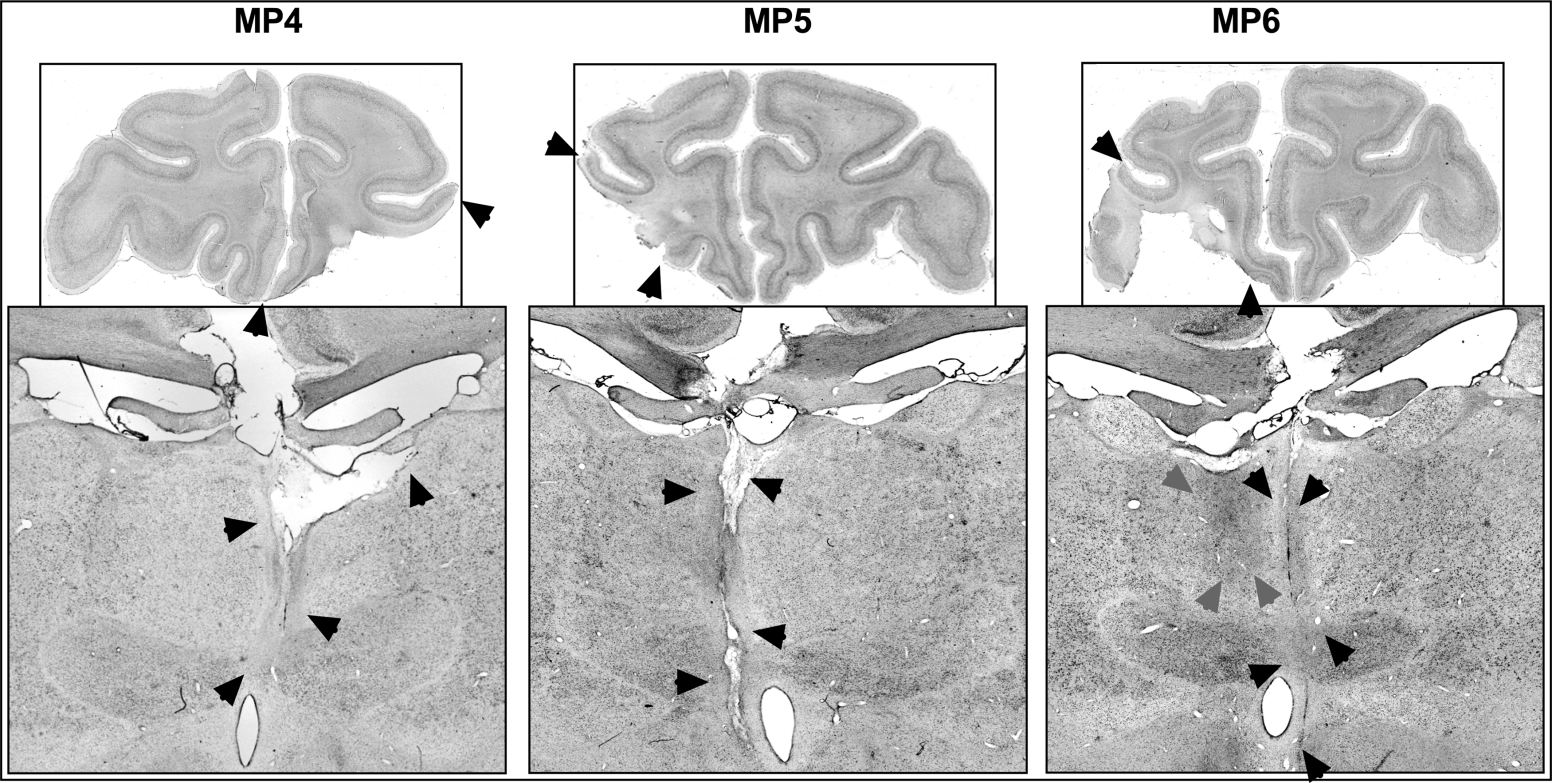
Top: Photomicrographs of a coronal slice at the level of IA+31 showing the unilateral damage (black arrows) to PFv+o in monkeys, MP4, MP5, and MP6. Bottom: Photomicrographs of a coronal slice at the level of IA+6 from monkeys MP4 and MP5 with ipsilateral hemisphere disconnection, showing the unilateral damage (black arrows) from the MDmc neurotoxic injections. Photomicrograph of a coronal slice at the level of IA+6 from animal MP6 with contralateral hemisphere disconnection, showing the unilateral damage (black arrows) from the midline neurotoxic injections and retrograde degeneration (gray arrows) caused by crossed PFv+o ablations in the ipsilateral hemisphere of MD.

Five of the 6 monkeys had unilateral damage to MDmc as intended. MP4 sustained damage to the MD pars caudodorsalis in addition (see Ray and Price 1993). Monkey MP6 did not sustain MDmc damage instead sustained a unilateral lesion to medial and ventral parts of the midline thalamic nuclei. Figures 2 and 4 show schematic drawings of the 6 medial thalamic lesions (and retrograde degeneration to neurons within MDmc after ablations in the contralateral PFv+o). Figure 3 shows photomicrographs of cresyl violet-stained coronal sections of the unilateral damage in MDmc and PFv+o (and retrograde degeneration in MDmc after the unilateral PFv+o ablation) for MP1, MP2, and MP3 with a contralateral disconnection of MDmc–PFv+o. Figure 5 shows photomicrographs of the cresyl violet-stained coronal section of MDmc and PFv+o for MP4 and MP5 with an ipsilateral disconnection of MDmc–PFv+o and MP6 with the midline thalamic lesion and contralateral disconnection of PFv+o. All monkeys also had sagittal section of the splenium of the corpus callosum dorsal to the posterior thalamus. This removal of splenium does not affect performance on the object-in-place scene discrimination task (Parker and Gaffan 1997b) and as evidenced by intact new learning in the operated midline thalamic lesion animal, MP6 (Table 1) (Figs 4–5).

### Assessment of PFv+o Lesions

The unilateral lesions of PFv+o included the intended cortical areas. All monkeys except MP3 had some sparing of Brodmann area 45/47 posteriorly. Brodmann area 13 (lateral orbitofrontal cortex) was spared in MP5. Figures 2–4 show schematics and photomicrographs of cresyl violet-stained coronal sections displaying the extent of the unilateral PFv+o lesions in all monkeys.

#### Object-in-Place Scene Discrimination Learning after Unilateral Lesions

**Figure 6.**
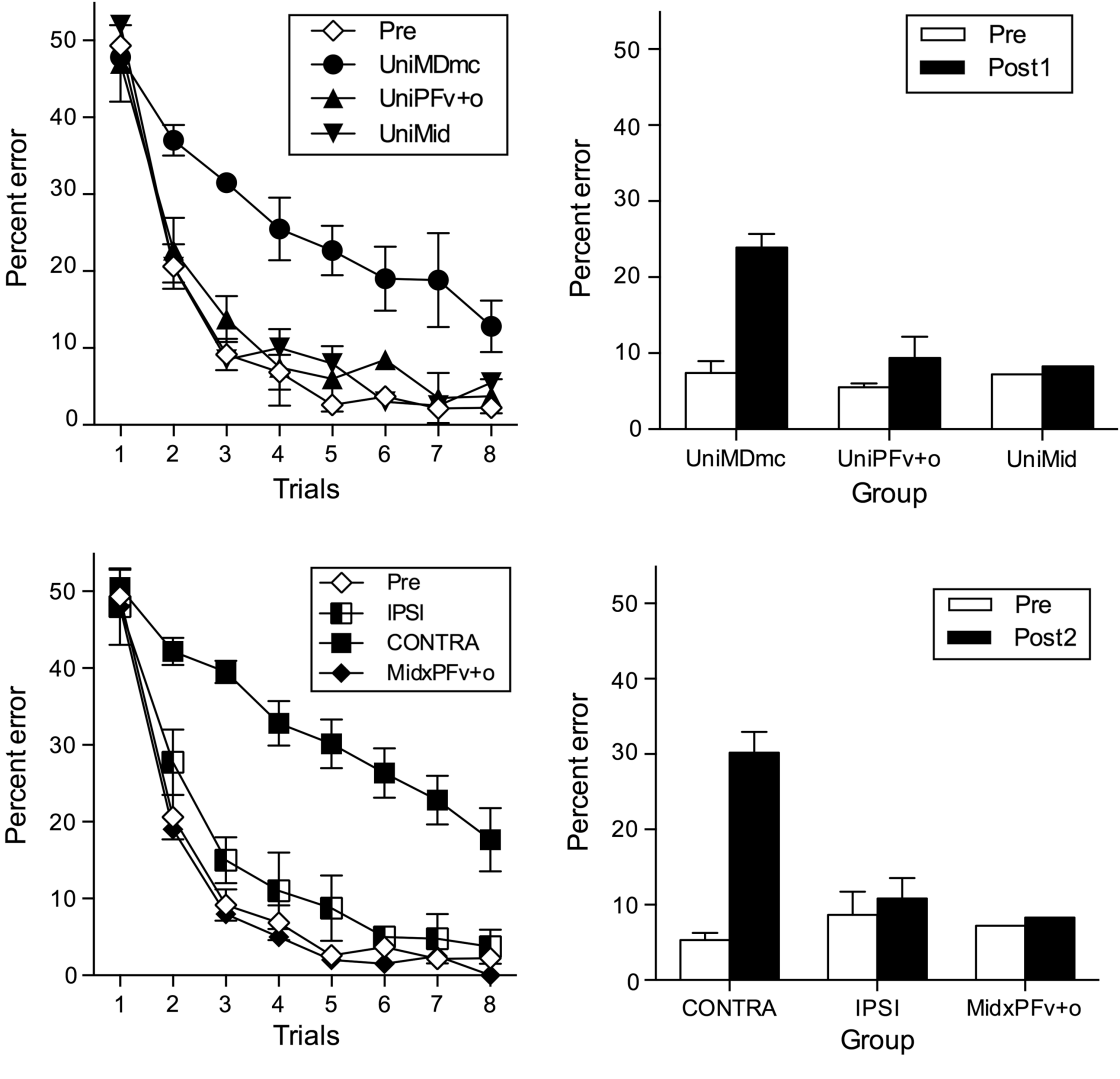
Object-in-place scene discrimination learning. Top left: preoperative (Pre) and postoperative mean percent error (+SEM) across the 8 repetition trials during the performance tests after the first unilateral neurosurgery for the groups of monkeys that received unilateral neurotoxic lesions to the medial, magnocellular subdivision of the mediodorsal thalamus (UniMDmc, *n* = 3) or unilateral ablations to the ventrolateral and orbital subdivisions of prefrontal cortex (UniPFv+o, *n* = 2) or unilateral midline thalamic damage (UniMid, *n* = 1). Top right: total mean (+SEM) percent error during the preoperative and postoperative (Post1) performance tests for the 3 lesion groups. Bottom left: preoperative (Pre) and postoperative mean percent error (+SEM) across the 8 repetition trials for the groups of monkeys that received contralateral hemisphere disconnection to MDmc–PFv+o (CONTRA, *n* = 3) or ipsilateral hemisphere disconnection to MDmc–PFv+o (IPSI, *n* = 2) or unilateral midline thalamic damage and contralateral disconnection of PFv+o (MidxPFv+o, *n* = 1). Bottom right: total mean (+SEM) percent error during the preoperative and postoperative (Post2) performance tests after the second neurosurgery for the 3 lesion groups.

The dependent measure was the mean percent errors made during learning new object-in-place scene discriminations repeated 8 times per session across 10 testing sessions during the preoperative and postoperative performance tests. The data from these tests are shown in Figure 6 (top, left, and right) and Table 1. Unilateral lesions to MDmc (UniMDmc, *n* = 3) impaired postoperative learning of object-in-place scene discriminations, with these monkeys making almost 3 times as many errors compared with preoperative performance. A repeated-measures *t*-test of the errors made during the preoperative (Pre) and postoperative (Post1) performance tests for the UniMDmc group confirmed that the additional errors made during learning after the first unilateral surgery (M = 23.90, SD = 5.33) were significantly different to preoperative performance (M = 7.41, SD = 4.59), [*t*_(2)_ = 34.74, *P* = 0.001]. In contrast, the errors made by the 2 animals with unilateral PFv+o ablations (UniPFv+o) during their postoperative performance test (M = 9.65, SD = 5.96) compared with errors made during their preoperative performance test (M = 5.54, SD = 0.95) suggests that the unilateral selective prefrontal ablations did not markedly disrupt their ability to learn new object-in-place scene discriminations [*t*_(1)_ = 1.16, *P* = 0.45]. Furthermore, the small change in errors made by the monkey (MP6) with UniMid neurotoxic damage during postoperative testing (M = 8.29) compared with preoperative testing (M = 7.21) suggests that for this midline thalamic damage, the surgical approach, the surgical procedure, and the recovery are not the cause of the deficits during learning for the UniMDmc monkeys.

#### Object-in-Place Discrimination Learning after Contralateral Hemisphere Disconnection

Given that the UniMDmc lesion caused animals to make significantly more errors during their postoperative performance test compared with their preoperative performance, it is important to note that the mean percent errors made after the second surgery to produce the contralateral disconnection (CONTRA) of MDmc in one hemisphere and the PFv+o ablation in the opposite hemisphere disconnection were greater (M = 32.29, SD = 4.45) for the 2 animals (MP1 and MP2) that received unilateral MDmc neurotoxic injections in the first surgery compared with errors made after their first neurosurgery (M = 21.07, SD = 2.93). A repeated-measures *t*-test indicates that this difference is significant [*t*_(1)_ = 10.43, *P* = 0.03]. Although there are only 2 animals in this analysis, it is suggestive that the CONTRA disconnection significantly adds to the deficits caused by the UniMDmc neurotoxic injections.

After the second surgery, monkeys (MP1, MP2, and MP3) from the CONTRA group were further impaired at learning object-in-place scene discriminations, making almost 6 times as many errors postoperatively. A repeated-measures *t*-test comparing the errors made during the preoperative performance test (M = 5.33, SD = 1.65) and second postoperative (Post2) performance test (M = 30.52, SD = 4.70) for the CONTRA group were significantly different [*t*_(2)_ = 9.14, *P* = 0.01]. In contrast, learning remained intact after ipsilateral disconnection (IPSI) of MDmc in the same hemisphere as the PFv+o ablations (this disconnection spared the intra-hemisphere connections in one hemisphere). The errors made by the 2 monkeys (MP4 and MP5) in the IPSI group during the second postoperative performance test (M = 10.86, SD = 4.65) were only marginally greater than the errors made during their preoperative performance test (M = 8.65, SD = 5.35), suggesting that the ipsilateral disconnection did not markedly disrupt their ability to learn new object-in-place scene discriminations [*t*_(1)_ = 4.42, *P* = 0.14] (Fig. 6, bottom, left, and right, and Table 1). The mean errors made by the monkey, MP6, with contralateral disconnection of midline thalamus from PFv+o (MidxPFv+o) during postoperative testing (M = 5.43) compared with preoperative testing (M = 7.21) suggests that contralateral midline thalamus and prefrontal cortex interactions are not critical for supporting learning of object-in-place scene discriminations.

#### Object Discrimination Learning and Reinforcer Devaluation

Monkeys with CONTRA or IPSI disconnection learned the rewarded object discrimination problems at a similar rate (mean sessions to criterion [not including the criterion run] [±SD] for each group: CONTRA, 15 [±8.19]; IPSI, 13.00 [±0]; mean errors to criterion [not including the criterion run] for each group: CONTRA, 221.33 [±76.58]; IPSI, 158.5 [±10.6]). Nonparametric Mann–Whitney *U*-independent *t* test confirmed that these differences were not significant (*P*s > 0.05). These errors and sessions to criterion were very similar to unoperated control monkeys from a previously published study (Mitchell, Browning et al. 2007) that learned the exact same stimuli in this object discrimination task (*n* = 4, errors M = 15.75 [±9.07] and sessions, M = 248.75 [±136.87]).

The performance data on the 2 devaluation tests from the monkeys with IPSI and CONTRA hemisphere disconnections are presented in Table 2. The difference scores were the main dependent measure of reward devaluation performance, with smaller positive difference scores representing attenuated decision-making after food value (satiety) devaluation. The difference scores were calculated as the difference in the number of choices of objects paired with a particular reward on the baseline sessions and in sessions when that food reward was devalued. These scores were added together for each devalued food during the critical test sequences for each test. The overall mean difference score was the average of the difference scores on Test 1 and Test 2. The difference scores were higher for the second devaluation test (M = 13.6, SD = 6.02) than the first (M = 6.8, SD = 4.87), which is congruent with the previous investigations using this task (Malkova et al. 1997; Baxter et al. 2000; Izquierdo and Murray 2004; Izquierdo et al. 2004; Mitchell, Browning et al. 2007). A nonparametric Wilcoxon Signed Rank repeated-measures *t* test using the entire sample of monkeys (CONTRA and IPSI) confirmed that the mean difference scores for the 2 devaluation tests were not significant (*Z* = 1.76, *P* = 0.078). Furthermore, there were no significant differences, when difference scores for the 2 devaluation tests were considered for each lesion group separately (*Z* < 1.34, *P* > 0.18).

**Table 2:** Food reinforcer devaluation performance (Experiment 2)

	Food devaluation Test 1				Food devaluation Test 2				
	Baseline M:P	Satiate M M:P	Satiate P M:P	Different score	Baseline M:P	Satiate M M:P	Satiate P M:P	Different score	Overall Mean
MP1	8:22	1:29	13:17	12	6.5:23.5	5:25	15:15	10	11
MP2	16:14	16:14	18:12	2	18.5:11.5	13:17	19:11	6	4
MP3	9.5:20.5	9:21	14:16	5	15:15	1:29	16:14	15	10
CONTRA Mean	11.2:18.8	8.6:21.4	15:15	6.33	13.3:16.7	6.3:23.7	16.7:13.3	10.33	8.33
MP4	26.5:3.5	17:13	29:1	12	26:4	8:22	30:0	22	17
MP5	28.5:1.5	27:3	30:0	3	29.5:0.5	15:15	30:0	15	9
IPSI Mean	27.5:2.5	22:8	29.5:0.5	7.5	27.8:2.2	11.5:18.5	30:0	18.5	13

Note: Baseline M:P refers to the number of M&M rewards and number of peanut rewards selected during the baseline critical test. Satiate M refers to the number of M&M rewards selected after satiation with M&Ms just prior to the critical test. Satiate P refers to the number of peanut rewards selected after satiation with peanuts just prior to the critical test.

**Figure 7.**
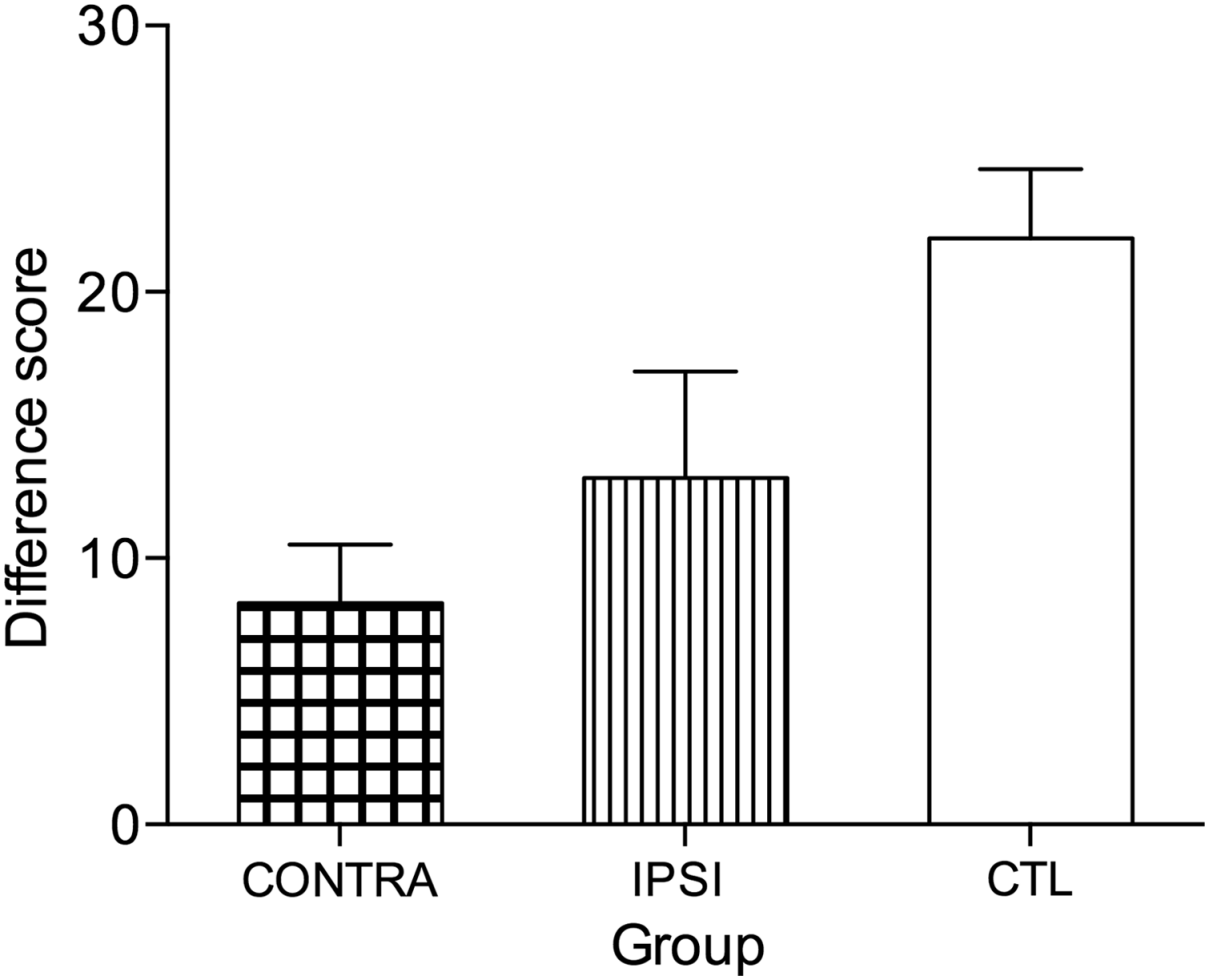
Food reinforcer reward devaluation. Overall difference score (+SEM) for the 2 reward devaluation tests for the 2 lesion groups, contralateral hemisphere disconnection to MDmc–PFv+o (CONTRA, *n* = 3) and ipsilateral hemisphere disconnection to MDmc–PFv+o (IPSI, *n* = 2). Unoperated control monkeys (CTL, *n* = 4) from a previously published study (Mitchell, Browning et al. 2007) are included for comparison.

Subsequently, the overall mean difference score for both tests were compared across the 2 groups (CONTRA and IPSI) as well as the difference scores for the 2 tests separately. While all monkeys showed intact learning of the discrimination problems that formed the critical trials for the devaluation test, monkeys with CONTRA (M = 8.33, SD ± 3.79) and IPSI (13.00, ±5.66; see Fig. 7) showed attenuated devaluation scores. By comparison, the overall mean difference score from the previously published unoperated control monkeys (Mitchell, Browning et al. 2007) showed that they did devalue the food reward that had been satiated just prior to the critical test sessions (M = 22.0, ±5.18). The overall mean difference score from the 2 tests of devaluation for the 2 groups (IPSI and CONTRA) were compared using nonparametric Mann–Whitney *U*-independent samples *t*-test, and the results showed that there was no difference between the 2 groups (*P* > 0.05). The separate analyses conducted for Test 1 and Test 2 were also not significant (*P*s > 0.05).

**Figure 8.**
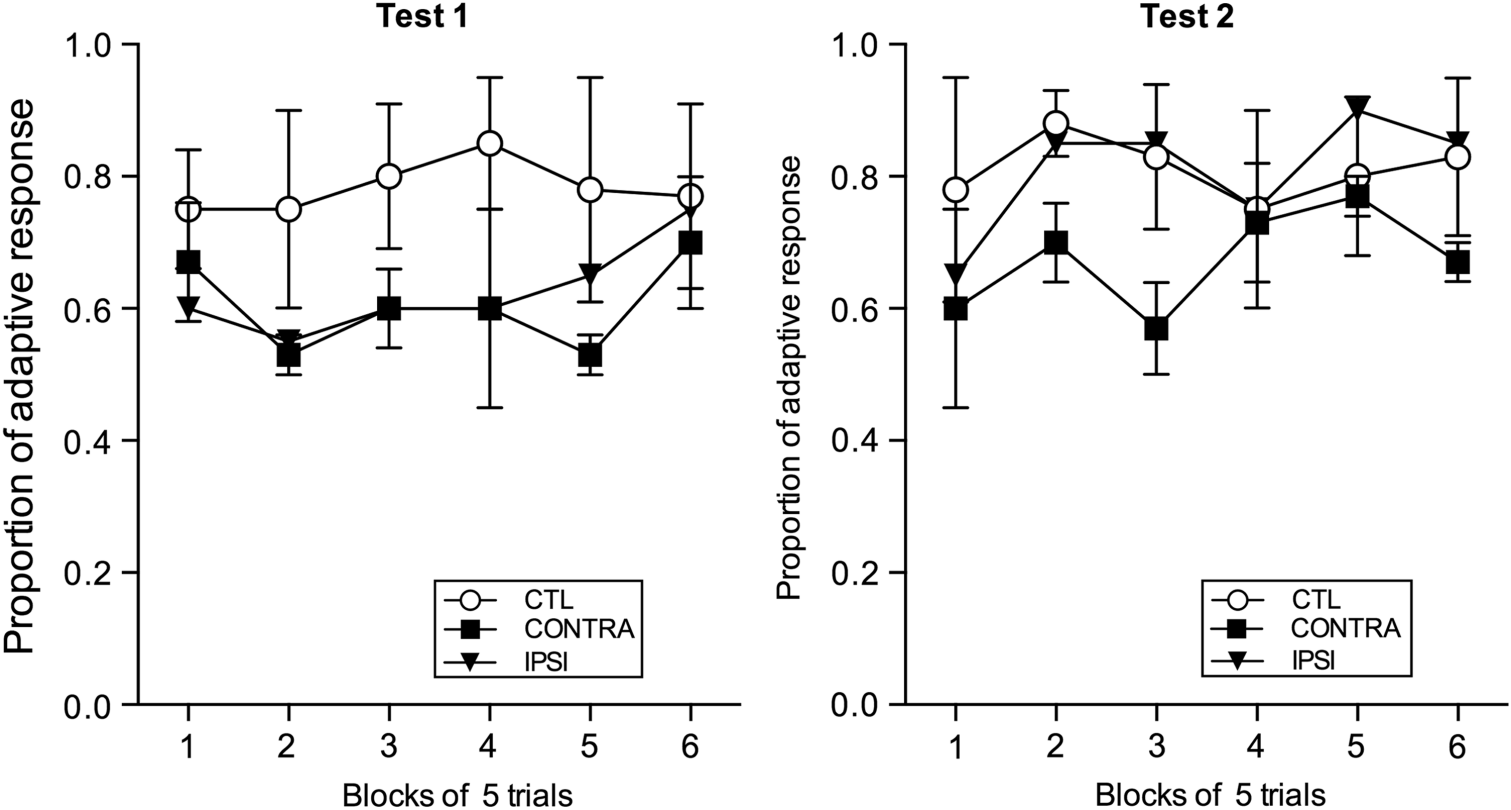
Adaptive responses (+SEM) during food reward devaluation for the lesion groups, contralateral hemisphere disconnection to MDmc–PFv+o (CONTRA, *n* = 3), and ipsilateral hemisphere disconnection to MDmc–PFv+o (IPSI, *n* = 2). Data from previously published unoperated control monkeys (CTL, *n* = 4) are included for comparison; these data were not previously presented in Mitchell and Browning et al. (2007).

To further explore possible group differences, the proportion of adaptive responses on the critical test sessions for each group (CONTRA and IPSI) was analyzed. Figure 8 shows the proportion of adaptive responses after selective satiation for Test 1 and Test 2, respectively. This measure, unlike the difference score, is independent of the baseline data. To compute the proportion of adaptive responses, each trial of the 30-trial devaluation test that followed the selective satiation was scored with either 1 or 0. A score of 1 was recorded when the chosen object was associated with the non-satiated food, whereas a score of 0 was recorded when the chosen object was associated with the devalued food. Thus, the monkeys with more adaptive responses had higher difference scores. Data from the 2 sessions (1 after devaluation of each food type) within each test were averaged and then divided into 6 blocks of 5 trials each. A 6 (trial block: 1, 2, 3, 4, 5, and 6) × 2 (group: CONTRA and IPSI) mixed-model repeated-measures ANOVA assessed the adaptive responses in each test and revealed no main effect of group for Test 1 (*F* < 1.0) or for Test 2 (*F* < 1.0). Thus, the proportion of adaptive responses shows that the monkeys with CONTRA and IPSI lesions were performing at a level indistinguishable from each other on the food reward devaluation task.

During Test 1 and Test 2, the groups consumed similar amounts of the devalued foods (Test 1 means [±SD]: CONTRA, 78.7 g [±23.19]; IPSI, 81.8 g [±8.13]) and (Test 2: CONTRA, 77.7 g [±57.7]; IPSI, 75.0 g [±0.77]). Repeated-measures ANOVA confirmed that the amounts consumed during the satiation procedures did not differ between the groups (*F*s < 1.0). All monkeys spent an average of 16.1 min in the devaluation test sessions.

## Discussion

This current study assessed cognitive abilities, including learning new object-in-place scene discriminations, learning object discriminations, and adaptive decision-making in monkeys who received either unilateral MDmc neurotoxic lesions or unilateral PFv+o ablation lesions and then after either combined contralateral or ipsilateral hemisphere disconnection of MDmc and PFv+o. These experiments produced several important findings and provide the first causal evidence in primates to support the hypothesis that interactions between the MDmc and PFv+o are critical for learning object-in-place scene discriminations and adaptive decision-making. First and surprisingly, our results demonstrated that unilateral neurotoxic MDmc lesions following the first neurosurgery are sufficient to produce deficits during learning as seen in the object-in-place scene discrimination task when compared with preoperative performance. Contrastingly, selective unilateral cortical lesions to PFv+o, like unilateral lesions to several other brain regions in primates (Easton and Gaffan 2001; Wilson et al. 2007), leave new learning intact on this task. Another study has also confirmed a lack of deficits after unilateral lesions to PFv+o when monkeys are required to rapidly learn conditional visuomotor associations (Bussey et al. 2002). Such dissociable behavioral results show that MDmc provides a unique functional role during learning new object-in-place scene discriminations, one that is different from its interconnected cortical targets. Interestingly, our result of the unilateral MDmc deficit in learning also contrasts with the results from a previous monkey lesion study, in which unilateral MDmc ablations did not impair object recognition memory (Parker and Gaffan 1998). Discussion of these contrasting results will be extended later.

Second and as predicted, the results demonstrate the functional importance of interactions between MDmc and PFv+o for learning new object-in-place scene discriminations. Monkeys with contralateral hemisphere lesions of the MDmc and PFv+o that disrupted information transfer in both hemispheres caused cognitive impairments whereas ipsilateral hemisphere disconnection (a lesion surgery that leaves one hemisphere functioning) left learning intact. These 2 results together suggest that MDmc and PFv+o contribute different but complementary roles during learning new object-in-place scene information, as will be discussed later. Third, the evidence of intact learning of object discrimination problems in Experiment 2 demonstrates that all forms of learning are not impaired after damage to MDmc and PFv+o. Similar intact object discrimination learning has also been reported after bilateral damage to either MDmc, orbital frontal cortex, ventrolateral, or dorsolateral prefrontal cortex using the exact same paradigm (Baxter et al. 2007, 2008, 2009; Mitchell, Browning et al. 2007). However, our results appear in contrast with other studies demonstrating deficits in object discrimination learning after bilateral MDmc damage (Gaffan and Murray 1990; Gaffan and Watkins 1991; Gaffan and Parker 2000). Reasons for the differences in these results will be discussed later. Finally, during the critical trials of reward devaluation, monkeys in both lesion groups had attenuated food reward devaluation scores highlighting how the integrity of the MDmc–PFv+o interactions is important for supporting monkeys to make adaptive choices associated with reward devaluation (Mitchell, Browning et al. 2007; Izquierdo and Murray 2010).

The current study combined evidence from 2 surgical lesion groups (contralateral and ipsilateral hemisphere disconnection). The ipsilateral hemisphere disconnection represents the best possible control procedure in a disconnection study because the 2 groups were identical except that the ipsilateral group spared the majority of intra-hemisphere connections. Therefore, we were able to compare the overall effect of the same type of surgery on performance across the 2 groups. If the surgery alone had caused the deficits, then differences between the 2 disconnection surgery groups would have been negligible. Furthermore, the dissociable results—intact learning after ventral midline thalamic neurotoxic injections compared with deficits after MDmc neurotoxic injections—suggests that the neurosurgical approach alone is not responsible for the extent of the cognitive deficits observed in the UniMDmc group. In another monkey lesion study, Gaffan and Murray (1990) removed midline thalamic structures via ablations in 3 monkeys in their group MD/A after the first surgery and these animals were also unchanged in their learning abilities. Finally, comparable surgical approaches have also been used on other operated control monkeys in different laboratories who subsequently demonstrated no impairments on cognitive testing in the object-in-place scene discrimination task (Parker and Gaffan 1997b; Izquierdo and Murray 2010).

Recent evidence shows that ablation lesions or neurotoxic injections into the orbital frontal cortex produce comparative deficits in the reward devaluation paradigm (Rudebeck et al. 2013). Unilateral lesions to PFv+o in our study were produced by ablations, and hence, white matter tracts within this cortical region are likely to have been damaged. However, we believe that our results of the monkeys' attenuated food reward devaluation performance are valid as the deficits demonstrated in our lesion groups were similar to those deficits demonstrated in both groups of monkeys with damage caused by either ablations or neurotoxic injections within the orbital frontal cortex (Rudebeck et al. 2013). Thus, both their study and ours provide causal evidence for the necessity to have integrity of neural interactions involving MDmc and orbital frontal cortex in reward-devalued decision-making paradigms in primates (Murray and Rudebeck 2013). Our current findings showing attenuated food devaluation scores in both CONTRA and IPSI hemisphere disconnected animals are comparable with previously published data in monkeys with bilateral neurotoxic lesions to MDmc using the exact same computerized paradigm (Mitchell, Browning et al. 2007). Furthermore, it is interesting that the 2 monkeys with the IPSI disconnection (MP4 and MP5) also had smaller, positive difference scores that were within the range of difference scores from the CONTRA lesion groups, and the differences in their means were not significant. Thus, our current results provide unique causal evidence to show that the interactions between MDmc and PFv+o are important for successful performance on this adaptive decision-making task in macaque monkeys. Complementary evidence from other monkey lesion studies has demonstrated that combined unilateral hemispheric damage to the amygdala and orbital frontal cortex is sufficient to cause attenuated food devaluation effects as well (Izquierdo and Murray 2004).

Critically, as indicated from the results of the reward devaluation paradigm, no one particular brain structure is by itself the crucial locus for the cognitive deficits, and from our behavioral evidence, this pertains to learning as well. The dissociable results showing learning deficits after unilateral MDmc neurotoxic lesions contrasted with intact learning after unilateral midline neurotoxic lesions or unilateral PFv+o ablations further extends our knowledge about the important additional contribution provided by MDmc during learning object-in-place scene discriminations. It has previously been shown that with bilateral removal of MDmc, learning but not retention of object-in-place scene discriminations is disrupted, indicating the more critical role of the MDmc in learning rather than retention of previously acquired information (Mitchell, Baxter et al. 2007; Mitchell and Gaffan 2008). Our current causal evidence therefore indicates that interactions between MDmc and the prefrontal cortex are important in supporting the acquisition of rapidly learned visual memories. Complementing these conclusions are cortical disconnection lesion studies in monkeys that signify the integrity of the prefrontal cortex is important for the acquisition and integration of visual information on a trial-by-trial basis (Browning and Gaffan 2008a; 2008b; Wilson and Gaffan 2008). Furthermore, a recent monkey electrophysiology study of lateral prefrontal cortex neurons during visual cognitive tasks (involving processing of objects, context, and location) suggests that attentional coherence within these neurons is important for organizing cognition (Kadohisa et al. 2015).

Other intriguing evidence from our study demonstrates that the MDmc–PFv+o interactions are not important for acquiring all new visual information. For example, monkeys showed learning deficits in object-in-place scene discrimination learning (Experiment 1), but object discrimination learning was left intact (Experiment 2). Bilateral neurotoxic MDmc damage also left acquisition of object discriminations intact using the exact same task (Mitchell, Browning et al. 2007). However, other studies do report impaired object discrimination learning after bilateral MDmc damage in monkeys (Gaffan and Murray 1990; Gaffan and Watkins 1991; Gaffan and Parker 2000). In these studies by Gaffan and colleagues (Gaffan and Murray 1990; Gaffan and Watkins 1991; Gaffan and Parker 2000), each pair of objects was presented several times within each learning session. In contrast, in the current object discrimination task, monkeys learnt the same 60 pairs of object–reward associations concurrently “across” sessions with each pair of objects presented only once in each session. Thus, in object discrimination tasks, when presentations of associations are made across sessions, monkeys with bilateral MDmc damage (Mitchell, Browning et al. 2007) or CONTRA or IPSI damage to MDmc–PFv+o can learn these object–reward associations as well as unoperated control monkeys.

Furthermore, during learning in the object-in-place scene discrimination task, multiple cognitive processes are required to be combined together for successful performance. The task requires that information about the object and its location on the screen is integrated together with reward in order to successfully complete the task. The colorful background “scene” provides additional context information. Associations of multiple cognitive attributes are necessary for successful performance as evidenced by the array of selective surgical lesions to different forebrain structures that cause deficits in the task (Gaffan 1994; Murray et al. 1998; Parker and Gaffan 1997a; 1997b; Gaffan and Parker 2000; Gaffan et al. 2001; Charles et al. 2004; Browning et al. 2005; Baxter et al. 2007, 2008; Mitchell, Baxter et al. 2007; Wilson et al. 2007, 2008; Mitchell et al. 2008). In relation to the mounting evidence showing the selective learning deficits observed after damage to MDmc, it is proposed that MDmc relays signals to the prefrontal cortex that support the prefrontal cortex in its role to integrate multiple cognitive attributes of task relevant information together on a trial-by-trial basis (Mitchell 2015). The inputs from the perirhinal cortex to MDmc (Russchen et al. 1987; Saunders et al. 2005) may also be important (Mitchell et al. 2014; Mitchell 2015) given the proposed role of perirhinal cortex in representing conjunctions of features (Bussey et al. 2005; Buckley and Gaffan 2006), although there are direct connections between the perirhinal and prefrontal cortex as well (Carmichael and Price 1995; Rempel-Clower and Barbas 2000).

These proposals are supported by some cross-species cognitive and behavioral studies. For example, patients with unilateral stroke damage that includes the MD display some forms of learning deficits and complex associative recognition memory impairments but not gross amnesia (Aggleton and Shaw 1996; Van der Werf et al. 2003; Pergola and Suchan 2013). Similar conclusions have been proposed for a “binding deficit” of spatial and temporal context information in patients with Korsakoff's syndrome, who have profound amnesia linked to widespread brain damage that includes the MD and PFC (Mayes and Downes 1997; Kessels and Kopelman 2012). Furthermore, in the rodent literature, similar dissociable results during learning of multiple cognitive processes versus object recognition are reliably found after damage to the MD (Mitchell and Dalrymple-Alford 2005; Cross et al. 2012) or MD-prefrontal cortex interactions (Cross et al. 2012). Taken together, this complementary cross-species evidence further suggests that the MDmc and MD-PFC interactions support the prefrontal cortex in its role to integrate multiple cognitive processes together during rapid learning.

Anatomical evidence suggests that the MD provides a critical link of communication within the frontal lobes via its indirect trans-thalamic route of signal transfer (Guillery 1995; Sherman and Guillery 2006, 2013; Mitchell et al. 2014; Mitchell 2015). Interestingly, an anatomical tracing study in primates has reported crossed as well as ipsilateral reciprocal connections exist between the MDmc and prefrontal cortex (Preuss and Goldman-Rakic 1987). Consequently, unilateral brain damage may be disrupting information transfer within both hemispheres as observed after the unilateral MDmc damage. However, we did not observe similar deficits in the IPSI group on the object-in-place scene discrimination task. One possible explanation may be that more widespread deficits occur with the loss of MDmc if the prefrontal cortex remains intact, than if it is also removed in the ipsilateral hemisphere as the MDmc damage. Another explanation may be that some recovery of function occurred over time in the animal (MP4) with the unilateral MDmc lesion carried out during the first operation. However, neither explanation was explicitly tested in this experiment. Nevertheless, our current cognitive and behavioral evidence further demonstrates that the MDmc and its interactions with the prefrontal cortex provide an important conduit for relay of signals important during learning of the object-in-place scene discrimination task (which requires rapid learning within a session) and adaptive decision-making (within a session). Conversely, the MDmc and PFC interactions do not seem as critical during certain types of learning (i.e., object discrimination learning [Experiment 2], which is learnt slowly across sessions) or during memory retention of familiar object-in-place scene discriminations (Mitchell and Gaffan 2008).

However, as yet it is unclear what underlying mechanisms the MDmc is providing to the frontal lobes during cognition. Complementary electrophysiology evidence from the rodent literature suggests that during learning MD neuronal discharge patterns synchronized with PFC local field potentials (Parnaudeau et al. 2013), while temporary inactivation of the entire MD altered medial PFC synchrony and correlated well with task performance (Parnaudeau et al. 2013, 2015). Others have proposed that synchrony in cortical oscillations enhances information binding, with the greater the degree of synchrony in the cortex, the better the information transfer between synchronized structures (Schmid et al. 2012; Uhlhaas and Singer 2013). Further, Saalmann and colleagues have provided direct evidence from electrophysiology studies in monkeys that the pulvinar (a “higher order thalamic relay” of the visual system) is regulating synchrony of interconnected cortical areas according to attentional allocation during a visuospatial attention task (Saalmann et al. 2012). Further studies are necessary to determine the underlying mechanisms linked to the interactions between the PFC and MDmc during learning and decision-making.

To conclude, surgical unilateral damage to MDmc in macaque monkeys causes deficits in learning object-in-place scene discriminations. Furthermore, when MDmc is damaged in combination with contralateral interconnected regions of prefrontal cortex, more widespread deficits occur during learning of object-in-place scene discriminations and adaptive decision-making. However, object discrimination learning remains intact. This causal behavioral evidence suggests a functional role for the MDmc thalamic relay in facilitating the frontal lobes to integrate several threads of task relevant information together rapidly during complex cognitive processes. Growing evidence is highlighting the important roles provided by higher order thalamic relay nuclei (like the MD) in supporting the cortex in the control of cortical information transmission and cognitive processing via trans-thalamic routes of communication (Saalmann et al. 2012; Schmid et al. 2012; Sherman and Guillery 2013; Saalmann 2014). Further studies investigating the functions of cortico-thalamo-cortical interactions, like those of MD with its cortical targets, are critical to understanding the role of the cortex during normal cognition and behavior and may lead to more targeted treatments for many neuropsychiatric disorders.

## Funding

This work was supported by a UK Medical Research Council Career Development Award (G0800329 to A.S.M.). Funding to pay the Open Access publication charges for this article was provided by an RCUK Open Access Block Grant to University of Oxford.

## Notes

We thank S. Mason for training the monkeys, G. Daubney for histology, and C. Bergmann and Biomedical Services for veterinary and animal husbandry assistance. *Conflict of Interest*: None declared.
